# The elusive allure of a rapid host blood signature for tuberculosis disease

**DOI:** 10.1128/jcm.01289-23

**Published:** 2024-01-25

**Authors:** Paul K. Drain, Ronit R. Dalmat

**Affiliations:** 1 Department of Global Health, University of Washington, Seattle, Washington, USA; University of Manitoba, Winnipeg, Manitoba, Canada

## Abstract

A rapid host transcriptional signature cartridge could be a major advancement for tuberculosis diagnosis and treatment monitoring. In a recent study, M. Li, Y. Qiu, M. Guo, R. Qu, et al. (J Clin Microbiol 61:e00911-23, 2023, https://doi.org/10.1128/jcm.00911-23) conducted an evaluation of the Cepheid 3-gene assay (Xpert-MTB-HR) within a diagnostic case-control study in China. While the study provides a strong contribution for determining the value of the Xpert-MTB-HR assay for diagnostic accuracy and treatment response, further assay optimization and more prospective studies are necessary before adaptation into clinical practice.

## COMMENTARY

One hundred forty years after the identification of *Mycobacterium tuberculosis*, Tuberculosis (TB) remains a leading infectious cause of death worldwide. Although a vaccine and effective medications have been available for decades, providing accurate diagnostic testing that is not reliant on imperfect smear microscopy or slow lab-based cultures has proven challenging. Furthermore, sputum testing has shown to have limited value for monitoring treatment response, yet new therapeutic biomarkers have been slow to emerge.

In the early 2000s, Cepheid (Sunnyvale, Ca) developed a 90-minute cartridge-based molecular assay, which changed the vision for TB diagnostics and other infectious diseases. By 2017, the second-generation Xpert MTB/RIF Ultra cartridge was approved by the World Health Organization and has now been widely used in TB-endemic countries. Despite these major advancements, the development of non-sputum-based diagnostics assays has been elusive and yet remains major priorities for national TB programs.

Given the inflammatory nature of TB disease, a host mRNA transcriptional signature was identified to distinguish TB disease from other infections (1). Since then, several clinical validation studies and a randomized controlled trial have been completed, each one causing both disappointment and optimism (2 – 4). Meanwhile, Cepheid has developed a novel molecular cartridge to quantify the presence of three host genes—guanylate-binding protein 5 (GBP5), dual-specificity phosphatase 3 (DUSP3), and Krüppel-like factor 2 (KLF2)—based on prior research (5). If successful, the three-gene signature cartridge could represent another major step forward for TB diagnosis and treatment monitoring.

In a recent article (6), Li and colleagues conducted a comprehensive evaluation of the Cepheid 3-gene host response assay (Xpert-MTB-HR) within a diagnostic case-control study in China. The team enrolled adults (≥15 years) who had TB-related symptoms or abnormal chest radiography, as well as healthy controls. The Xpert-MTB-HR test was performed on 100 microliters of venous whole blood that was collected at the same time as the three-sputa sample. A “TB score” was calculated for the Xpert-MTB-HR result by subtracting the cycle threshold of KLF2, which is downregulated in TB, from 1/2 the added cycle threshold values for GBP5 and DUSP3, which are both upregulated in TB (5). After combining three sputa specimens, from those with signs or symptoms of TB, and performing some pre-analytical concentration (which is atypical for an Xpert assay), the resuspended specimen was tested by Xpert Ultra and liquid culture. The final cohort included 149 symptomatic adults with microbiologically confirmed TB disease, 248 symptomatic adults negative by Ultra and liquid culture (presumed other respiratory diseases), and 193 adults without signs or symptoms of TB as healthy controls.

Overall, the expression of the three genes, as well as a composite TB score, was significantly different between the TB disease and healthy control groups. Two of the three genes and the TB score were significantly different when comparing TB disease and other respiratory disease groups. When comparing participants with TB disease to the healthy controls, the Xpert-MTB-HR had good discriminatory value (AUC = 0.912). However, when comparing adults with TB disease to people with other respiratory infections, which is a more informative comparison group for the purposes of evaluating a triage test, the Xpert-MTB-HR had only modest discriminatory value (AUC = 0.798). In both comparisons, the discriminatory values were higher among persons with a higher *Mtb* bacillary load, as determined by the cycle threshold values of the Ultra result.

In addition, the team assessed changes in the TB score at 2, 5, and 6 months after the start of therapy to assess its value as an indicator of treatment response. While the average TB score was higher after 2 months of treatment, one-quarter of the patients had TB scores that declined during treatment, despite symptomatic improvement. Since the TB score was expected to increase with treatment, additional evaluations or testing was not completed to explain this paradoxical response.

The study provides a strong contribution for determining the value of the Cepheid Xpert-MTB-HR assay for diagnostic accuracy and treatment response. While there are some important results, they should be interpreted with caution. First, the recruitment of the healthy control group occurred outside the triage setting, and therefore, results of test performance in this group do not provide a comparison that is interpretable in real-world diagnostic settings. The performance characteristics determined from comparing results among individuals with TB-related symptoms or abnormal chest radiographs are more accurate, since comparing the TB disease group to a healthy control group will overstate the accuracy or discriminatory value of the Xpert-MTB-HR assay. Second, the study reported a very large range and distribution of the composite TB score for the TB disease cohort at baseline and during follow-up visits. The distribution of the TB score may reflect the imprecise nature of measuring mRNA. Further data on reliability of the assay, such as a triplicate testing among a subset of participants, could have helped define the within-person variability.

While the inflammatory response is strongly activated during TB disease, it is possible that the Cepheid cartridge may not be measuring the optimal signature. Others have reported different sets of genes that may have superior accuracy or treatment monitoring performance (7). Additional testing of inflammatory markers may help elucidate differences in the Xpert-MTB-HR assay results. In addition, inflammatory biomarkers generally recede within 2–4 weeks after starting therapy, and 2 months is a long period to wait for assessing the therapeutic response. Therefore, another study with more routine follow-up during the induction phase of treatment may provide more granular data on the host response signature for treatment response.

Overall, the results of this study may represent another “two steps forward, one step back” for transcriptomic testing for TB disease. The successful completion of a rapid host transcriptional signature would be a major advancement for the TB field. More importantly, a non-sputum-based assay to diagnose incipient TB or early subclinical TB could change prevention and treatment efforts to achieve the WHO’s END TB Strategy goals.

## References

[1] Berry MPR , Graham CM , McNab FW , Xu Z , Bloch SAA , Oni T , Wilkinson KA , Banchereau R , Skinner J , Wilkinson RJ , Quinn C , Blankenship D , Dhawan R , Cush JJ , Mejias A , Ramilo O , Kon OM , Pascual V , Banchereau J , Chaussabel D , O’Garra A . 2010. An interferon-inducible neutrophil-driven blood transcriptional signature in human tuberculosis. Nature 466:973–977. doi:10.1038/nature09247 20725040 PMC3492754

[2] Anderson ST , Kaforou M , Brent AJ , Wright VJ , Banwell CM , Chagaluka G , Crampin AC , Dockrell HM , French N , Hamilton MS , Hibberd ML , Kern F , Langford PR , Ling L , Mlotha R , Ottenhoff THM , Pienaar S , Pillay V , Scott JAG , Twahir H , Wilkinson RJ , Coin LJ , Heyderman RS , Levin M , Eley B . 2014. Diagnosis of childhood tuberculosis and host RNA expression in Africa. N Engl J Med 370:1712–1723. doi:10.1056/NEJMoa1303657 24785206 PMC4069985

[3] Zak DE , Penn-Nicholson A , Scriba TJ , Thompson E , Suliman S , Amon LM , Mahomed H , Erasmus M , Whatney W , Hussey GD , et al. . 2016. A blood RNA signature for tuberculosis disease risk: a prospective cohort study. Lancet 387:2312–2322. doi:10.1016/S0140-6736(15)01316-1 27017310 PMC5392204

[4] Warsinske H , Vashisht R , Khatri P . 2019. Host-response-based gene signatures for tuberculosis diagnosis: a systematic comparison of 16 signatures. PLoS Med 16:e1002786. doi:10.1371/journal.pmed.1002786 31013272 PMC6478271

[5] Sweeney TE , Braviak L , Tato CM , Khatri P . 2016. Genome-wide expression for diagnosis of pulmonary tuberculosis: a multicohort analysis. Lancet Respir Med 4:213–224. doi:10.1016/S2213-2600(16)00048-5 26907218 PMC4838193

[6] Li M , Qiu Y , Guo M , Qu R , Tian F , Wang G , Wang Y , Ma J , Liu S , Takiff H , Tang Y-W , Gao Q . 2023. Evaluation of the Cepheid 3-gene host response blood test for tuberculosis diagnosis and treatment response monitoring in a primary-level clinic in rural China. J Clin Microbiol 61:e00911-23. doi:10.1128/jcm.00911-23 37902328 PMC10662368

[7] Scriba TJ , Fiore-Gartland A , Penn-Nicholson A , Mulenga H , Kimbung Mbandi S , Borate B , Mendelsohn SC , Hadley K , Hikuam C , Kaskar M , et al. . 2021. Biomarker-guided tuberculosis preventive therapy (CORTIS): a randomised controlled trial. Lancet Infect Dis 21:354–365. doi:10.1016/S1473-3099(20)30914-2 33508224 PMC7907670

